# Patient safety culture as a space of social struggle: understanding infection prevention practice and patient safety culture within hospital isolation settings - a qualitative study

**DOI:** 10.1186/s12913-022-08703-x

**Published:** 2022-11-29

**Authors:** Julian Hunt, John Gammon, Sharon Williams, Sharon Daniel, Sue Rees, Sian Matthewson

**Affiliations:** 1 Faulty of Medicine, Health and Life Sciences, Swansea University, Swansea, Wales UK; 2 Hywel Dda University Health Board, Carmarthen, Wales UK

**Keywords:** Patient safety culture, Infection prevention, Patient safety, Focus group interviews, Bourdieu, Healthcare staff, Hospital, Hospital isolation, Healthcare, Wales

## Abstract

**Background:**

In recent times, infection prevention and patient safety have become a global health policy priority with thought being given to understanding organisational culture within healthcare, and of its significance in initiating sustained quality improvement within infection prevention and patient safety. This paper seeks to explore the ways in which engagement of healthcare workers with infection prevention principles and practices, shape and inform patient safety culture within the context of hospital isolation settings; and vice-versa.

**Research methods:**

In this paper, we utilise focus group interviews at two hospital sites within one health board in order to engage healthcare staff in elaborating on their understandings of infection prevention practices and patient safety culture within isolation settings in their organisation. Focus group transcripts were analysed inductively using thematic analysis in order to identify and develop emerging empirical themes.

**Results:**

Positioned against a background of healthcare restructuring and ever-increasing uncertainty, our study found two very different hospitals in regard to patient safety culture and infection prevention practice. While one hospital site embodies a mixed picture in regard to patient safety culture, the second hospital is best characterised as being highly fragmented. The utilisation of focus group interviews revealed themes that capture the ways in which interviewees position and understand the work they perform within the broader structural, political and cultural context, and what that means for infection prevention practice and patient safety culture.

**Conclusion:**

Drawing on the insights of Bourdieu, this paper theorises the field of patient safety as a space of social struggle. Patient safety is thus positioned within its structural, cultural and political context, rather than as merely an epidemiological dilemma.

## Background

Since the turn of the century, patient safety culture and the quality of care provided in the National Health Service (NHS) has come under significant scrutiny following a number of high profile cases; such as the cases of Bristol Royal Infirmary, Mid-Staffordshire NHS Hospital Trust and maternity services at the Shrewsbury and Telford Hospital NHS Trust and at Cwm Taf Morgannwg University Health Board in South Wales.^1^ Concerns regarding the safety of paediatric cardiac surgery at Bristol Royal Infirmary between 1984 and 1995, and of the high mortality rates of babies and safety of maternity services at Cwm Taf Morgannwg Health Board in 2019, led to two of the most far reaching public reviews ever untaken into the workings of the NHS. Both reports highlighted significant problems with the quality of patient experience, team working, communication and whistle blowing, accountability and organisational cultures [1, 2]. The Independent Review of Maternity Services at the Shrewsbury and Telford Hospital NHS Trust that commenced in the summer of 2017, highlighted the failures of maternity services within the Trust over two decades [3, 4]. The review identified a number of issues uncovered in previous scandals within the health service. These include: failures in leadership and teamwork, failures in following clinical guidelines, failures to listen to parents and patients, and failures to learn from mistakes and improve. Identified within the inquiry were ‘significant or major concerns’ relating to the care involved in nine maternal deaths, 131 stillbirths, 29 cases of hypoxic ischaemic encephalopathy (HIE), and 70 neonatal deaths; in addition to near 65 cases of brain damage that were often diagnosed only years later. Weaknesses in leadership and teamwork, identified in prior investigations into failings in maternity services at Furness General Hospital, Morecambe Bay [5] and at Cwm Taf Morgannwg Health Board in South Wales [2], included a culture of bullying and repeated failures by the board in facing up to problems. These reports have and continue to inform the UK patient safety agenda and help frame a broader policy agenda that may have been more difficult had the existing landscape of professional and organisational regulation not been shown to be lacking.

Patient safety practices are crucial in healthcare, particularly in terms of the prevention of infections and as identified in the national patient safety campaign, *Sign Up To Safety*, is considered as the responsibility of patients and service users; as well as practitioners. Patient safety has been defined as ‘the avoidance, prevention and amelioration of adverse outcomes or injuries stemming from the process of healthcare’ [6] and throughout the first two decades of the twenty first century, has become a global health policy priority; highlighted most recently with the COVID-19 pandemic. These years have seen the introduction of a number of strategies to support organisational learning [7–11]. That is, learning characterised as a continuous cycle of action and reflection [12]. The Public Enquiry into Mid-Staffordshire NHS Trust [13] highlighted the systemic failure to learn from and respond to unsafe patient care. The report demonstrated the ways in which hospitals experience difficulties in maintaining focus on patient safety practices, becoming preoccupied with the business of the system (finances and targets) rather than the quality and safety of patient care. The subsequent Berwick Report generated lessons and suggestions for change for the UK government and the NHS in England [14]. Nevertheless, reforms to enhance patient safety have proved challenging to implement; as recent reviews into maternity services Cwm Taf Morgannwg University Health Board [2] and Shrewsbury and Telford Hospital NHS Trust [3, 4] signify.

Infection prevention occupies a unique position within the field of patient safety in that it is universally relevant to nursing staff, other healthcare workers and patients at every healthcare encounter. In this way, the infection prevention focus of the Ebola outbreak response in West Africa 2014–2016, was critical in preventing transmission, both within communities and healthcare facilities through the implementation of infection prevention practices and context specific strategies. In similar ways, infection prevention measures have been established for the early detection, containment, delay and control of the global pandemic of Coronavirus (COVID-19) that was initially identified in Wuhan, China in late December, 2019 causing severe respiratory disease including pneumonia. Following the initial outbreak, COVID-19 specific infection prevention measures were rapidly introduced and include:

Barriers: Isolation, in negative pressure single room if available, of confirmed COVID-19 cases and of individuals meeting the COVID-19 case definition awaiting test results.

Barrier precautions: Personal protective equipment (PPE), screening and testing, and self-isolation.

Skin: Hand hygiene, skin decontamination, cleansing and antisepsis.

Environmental infection prevention: Respiratory and cough hygiene, environmental decontamination, and the role of the environment and equipment.

Effective reporting within and between healthcare organisations, public health agencies and government.

Moreover, it is recognised that infection prevention practices and procedures, importantly, isolation precautions, are significant in preventing and controlling the transmission of COVID-19, MRSA, Norovirus, Clostridium difficile and any number of healthcare-associated infections (HCAIs). Thus the priority being to ensure effective infection prevention practices and procedures are positioned within a wider framework of quality improvement, and the safety of patients, nursing staff and other healthcare workers.

Since the turn of the twenty first century, thought has been given to understanding the shared attitudes, beliefs, values and assumptions that underlie peoples’ actions in regard to issues of safety; and of the potential importance of these shared characteristics in initiating sustained changes within infection prevention and patient safety [9, 15, 16]. Within the literature, these shared characteristics are often referred to as the ‘safety culture’ of an organisation [17]. The challenge being to create a culture of patient safety, ensuring that the responsibility for infection prevention is embedded at all levels of an organisation. The notion of ‘safety culture’ first emerged in 1988, following the 1986 nuclear energy Chernobyl disaster.

In offering a contribution to the debate around infection prevention and patient safety culture, this paper reports on a qualitative study of infection prevention practices and patient safety culture within isolation settings at two NHS hospitals in Wales, UK. The paper aims to explore the ways in which engagement of healthcare workers with infection prevention principles and practices, shape and inform patient safety culture within the context of hospital isolation settings; and vice-versa. We sought to understand perceptions of infection prevention ownership and responsibility for healthcare workers; the ways in which infection prevention practice is promoted, how infection prevention teams operate as new challenges arise and of the positioning of infection prevention practice within the broader context of organisational patient safety culture, within hospital isolation settings. There is an abundance of available evidence supporting the measurement of safety culture within mainstream healthcare settings [18]. In this paper, we utilise focus group interviews in order to engage nursing staff and other healthcare workers in elaborating on infection prevention practices and patient safety culture within their organisation. Drawing on the insights of Bourdieu [19, 20], we theorise the field of patient safety as a space of social struggle.

### Infection prevention and hospital isolation

In regard to infectious diseases, the notion of isolation refers to the possibility of separating infected people (or those suspected to be infected) from the broader population. The practice of isolation has historically been used in controlling and preventing the spread of infectious diseases and microorganisms such as COVID-19, MRSA, Clostridium difficile and Norovirus, and known routes of transmission of infection in healthcare facilities. The operation of standard precautions, including single room isolation, in addition, wherever necessary, to transmission-based precautions (TBPs), is understood as being a cornerstone of infection prevention practice, and is implemented for patients who are either known or suspected of being infected or colonised with pathogens spread through air, droplet or contact routes.

Although a seemingly simple notion, isolating patients is complex and challenging in implementation [21–23]. Caring for isolated patients fluctuates depending on the structure of the organisation, available resources and the changing epidemiology of HCAIs. Effective hospital isolation further involves nursing staff and other healthcare workers, and on occasions patients and visitors, conforming to particularly strict protocols. In addition to the prevention of occupational exposure to infectious diseases / pathogens via, for example, available immunisations, these protocols concern adherence to specific requirements of hospital isolation, the correct use of PPE, performing appropriate hand hygiene, the correct management of linen and waste, and the cleaning and decontamination of equipment and the environment; as well as. Moreover, each of these elements must be implemented without compromising the safety of patients. Even while research suggests that placing a patient in hospital isolation can have a serious impact on their wellbeing, welfare and liberty [21–23], the practice of creating safe spaces is based on a sound theoretical rationale and is critical for effective infection prevention [22].

### Theoretical positioning – patient safety culture as a space of social struggle

In understanding the culture of patient safety within hospital isolation settings, the theoretical insights of Bourdieu [19, 20] are particularly helpful. Bourdieu proposes a structural theory of practice which couples structure and agency in a dialectical relationship between structure, culture and power. He recognises the social relations among actors as being structured by, and in turn, contributing to the structuring of, the social relations of power among different positions. Bourdieu’s theoretical framework involves three primary notions. These are: Field, habitus and capital. For Bourdieu [19, 20], society contains a number of fields including healthcare, education and the state. The notion of field emphasises the dynamic relationships that give rise to social action within a given social space. Fields are understood as being bounded configurations or networks of ‘objective relations between positions’ [24]; identifiable by the shared concerns and activities of those within them. A field is a space of social struggles in which individuals and groups struggle for dominance over their field through acquiring and utilising forms of capital valued within that field. Bourdieu [25] defines four forms of capital circulating within fields. These are: Economic capital (financial resources), social capital (networks of valued relations), cultural capital (legitimated knowledge) and symbolic capital (honour and prestige). Capital in any given field is affected (For example: Valued, traded or ignored) by other fields and sub-fields. Bourdieu’s notion of field includes ideas, thoughts and actions; as well as the resources it attracts. The position of an individual within a field is governed by their access to resources and related power - The capital within that particular field. Each field or sub-field, such as healthcare and patient safety, will further have ‘its own orthodoxy, its own way of doing things, rules, assumptions and beliefs; in sum, its own legitimate means’ [26].

Social structures can thus be understood as being both objective and subjective. The acquisition and distribution of capital can be quantified and described objectively, while subjectively, the process leads those involved normalising the forms and distribution of capital. This forms part of a process of making sense. In turn, this process produces the individual’s habitus. That is, their internalised history. A person’s habitus involves a system of ‘durable, transposable dispositions, that are structured, inculcated and generative’ [19]; by which an individual understands and aligns to the social world. Experiences become embodied within the habitus and it is through these experiences people develop a ‘feel for the game’ [27]; learning rules that become second nature.

Habitus is the product of history and is conditioned by experience in the social structure [19]. People’s agency and sense of possibility are shaped by the habitus. While habitus provides form and coherence to a person’s relationship with one or any number of fields, Bourdieu draws on the notion of doxa to offer further understanding as to what it means to feel at home in a field. That is, where there is a close fit between the subjective aspects of habitus and the objective structures and rhythms of a social setting [28]. Doxa involves knowledge shaped by experience. In this way, doxa reflects the taken for granted ordering of a field that builds over time for the independent and interdependent experiences and interactions in familiar fields between people who share a similar habitus [29].

According to Bourdieu [24], the work of the social scientist is, in large part, to problematise the taken for granted nature of these constructions. In the past, research into patient safety primarily focused on the causes, consequences and rates of errors, and the strategies to reduce them. Thus patient safety has largely been theorised as an epidemiological problem or technical strategy [30, 31]. Drawing on the insights of Bourdieu, this paper attempts to understand the culture of patient safety as being a sub-field of the wider field of healthcare. In doing so, patient safety is positioned within its social, cultural and political context; as a space of social struggle, rather than as merely an epidemiological or technical dilemma.

## Research design and methods

The research explored infection prevention practice and patient safety culture within hospital isolation settings, with nursing staff and other healthcare workers at two District General Hospitals (DGHs) at one health board in Wales, UK. Analysing and understanding a hospital’s patient safety culture is a particular challenge [32–34], especially in terms of infection prevention practices within isolation settings. While there are both quantitative and qualitative approaches, there is no clear consensus as to which method is best for exploring safety culture in specific healthcare contexts.

The research conducted during 2018 and 2019, and prior to the COVID-19 pandemic, involved a series of two focus group interviews with healthcare professionals, including nursing staff, held four months apart, at each hospital site.

### Study setting

The health board serves both urban and rural populations across three counties and provides a full range of acute, intermediate, primary and community care services. Acute care is concentrated in four hospitals. The organisation employs 10,000 staff directly involved in patient care. At the time of fieldwork, the health board was going through a period of restructuring and what it termed ‘a radical shake-up’ to healthcare across this part of Wales, UK. The case study hospital sites reflect a range of demographic and organisational characteristics which may impact on infection prevention practice and patient safety culture within isolation settings.

Hospital Site A is the largest hospital within the health board, and is going through increasing uncertainty and resource constraints. As part of wider health board restructuring plans, Hospital A, along with a second hospital, will lose services and be repurposed. A new hospital, combining both hospitals, is to be built at a new location.

Hospital Site B serves a large, rural catchment area with particularly poor transport links. In recent times, its administration and future operation have been a subject of controversy with the hospital experiencing a substantial budget deficit and critical staffing shortage resulting in the temporary closure of beds. Focus group interviewees viewed these circumstances as a consequence of mismanagement and spoke of Hospital Site B in terms of being orphaned within the health board. This, in part, is due to the geographical separation of the hospital from the other three DGHs within the health board; as well as political positionings.

### Data collection - focus group interviews

Increasing recognition of the benefits of the qualitative research paradigm has opened up new means of exploration and investigation. In this way, there has been an increase in the use of focus group interviews as a viable alternative to traditional one-to-one interviews. Krueger identifies focus groups as being ‘a carefully planned series of discussions designed to obtain perceptions on a defined area of interest in a permissive, nonthreatening environment’ [35]. Focus groups offer semi-informal spaces with the aim being to elicit a discussion that allows the researcher to see the world from the participants’ perspectives in an open, free, relaxed format. Focus group discussions allow the researcher to probe both the intellectual reasoning and emotional responses of participants while observing the underlying group dynamic.

The focus group interviews were undertaken with a purposive sample of NHS workers at both hospital sites. In utilising purposive sampling [36], our aim was to obtain ‘rich data’ from a small number of healthcare workers, including nurses, selected because of their position and ability to provide in-depth information regarding the primary interest of our study. Our research design further utilised a variant of purposive sampling known as ‘maximum variability sampling’; in that our aim was to include a wide variety of different members of staff in terms of occupational group and seniority in order to explore the breadth of views and knowledge across occupational groups in regard to infection prevention practice and patient safety culture. The lead infection prevention nurse at each hospital site identified appropriate members of staff who they subsequently contacted and provided with letters of invite and study information sheets. Interviewees in our focus groups included a surgical consultant, junior doctor, senior nurses, nurses, healthcare support workers, hotel services / domestics, physiotherapists, a phlebotomist and an occupational therapist (See: Table 1). All had been employed within the NHS in excess of three years at the time of the focus group interviews taking place and all had job roles that involved the isolation of hospital patients.

**Table 1:** Focus Group Participants by Job Role

Hospital Site A	Hospital Site B
**Job Role**	**Job Role**
Physiotherapist Occupational Therapist Senior Staff Nurse Staff Nurse Staff Nurse Staff Nurse Hotel Services Hotel Services Healthcare Support Worker Healthcare Support Worker Junior Doctor	Healthcare Support Worker Hotel Services Hotel Services Hotel Services Phlebotomist Consultant – Orthopaedics Senior Staff Nurse Staff Nurse Staff Nurse Physiotherapist

The focus groups interviews were facilitated by the study researchers. Each focus group involved between 4 and 10 interviewees, and lasted for around 60 minutes. The focus group interviews took the form of open discussion. The open discussions were steered utilising a topic guide with open-ended questions, and were recorded with permission and transcribed verbatim. The focus groups explored a number of topics including interviewee understandings of infection prevention ownership and patient safety culture, and what this meant for the work they perform (See: Table 2).

**Table 2:** Topics Explored During Focus Group Interviews

Topic
Perceptions of working within the health board and at the hospital sites.
Staff education and training.
Learning and effecting change.
Understandings of infection prevention and patient safety.
Responsibilities for infection prevention at organisational and hospital ward levels.
Responsibilities and accountability for priority given to patient safety at organisational and hospital ward levels.
Challenges and support involved in providing effective infection prevention practices in terms of delivering quality of care.
Management and worker relations.
Leadership
Communication.
Collaborative working and team working.
Understandings of infection prevention ownership and patient safety culture, and what this means for the work focus group interviewees perform.

### Data analysis

Focus group transcripts were analysed inductively using thematic analysis in order to identify and develop emerging empirical themes. This approach involved a process of close reading, re-reading and noting initial ideas, systematically coding themes across our data, collating these initial codes into potential themes. The coding process involved breaking down data contained within the transcripts into discreet parts for closer examination and these were compared for consistency and coherence. Throughout this process, an analytic document was kept as each transcript was coded to keep track of thoughts and ideas, and to reflect on the coding process. Potential themes were further reviewed and refined until new data added no further conceptual insights. Extracts were then identified as exemplars of each theme. The process of coding was supported by the NVivo 12 computer software package.

Analysis of data and initial coding was led by the field researcher, with a second researcher coding samples of data to examine the consistency of codes and contributing to secondary coding. As data analysis progressed and in order to ensure adequate quality and validity of the qualitative analysis process, the analytical process, analytic document and emerging themes were shared, reviewed and discussed among members of the research team at different stages. This helped with clarification and the refining of analytic themes, which were cross-referenced with the literature. By comparing and contrasting the hospital sites and contexts, drawing on the work of Bourdieu, we sought to move beyond description of differences to offering theoretical insights into the social world of healthcare work.

NHS Research Ethics Service full ethical approval was received from Wales REC 7 on March 27, 2018 (REC reference: 18/WA/0113). Further approvals were obtained from the participating health board and participating hospital sites. All study interviewees were provided with written information regarding the study and their informed consent sought. Interviewee consent was obtained in writing. Participation was voluntary and interviewees were free to withdraw at any time.

## Results / main findings

### Hospital site A

The focus group interviews at Hospital Site A were notable for its commitment and focus to the patients staff serve; many of whom are older in age, from isolated rural villages and who speak the Welsh language as first language or sole language. This level of commitment to patients has, in a number of ways, established a shared vision for the site with staff members working together in making noticeable improvements to the healthcare environment and to ensure patients are cared for appropriately. This vision involves staff sharing knowledge and bridging cultures with teams across departments; often in challenging circumstances.

The strong culture and emotional commitment at Hospital Site A was tempered, however, by structural and political challenges. Within the past 18 months, there was the view that the hospital has experienced significant staffing shortages. Across the same period, there has been a renewed push within the organisation for continuous improvement. The resulting change in focus from management has increased the pace and rhythms of the hospital ward leaving staff feeling stretched and frustrated:

*‘It’s hard with any initiative to keep going when you have the challenges of day to day staffing (on the ward)’*.

In many ways, staff teams are feeling left behind within the organisation. While staff feel there is a largely positive safety culture across the organisation, their reality on the hospital ward is somewhat more mixed. The organisation has processes in place to share learning, such as reflection and sharing patient perceptions. Staff are actively involved in the process and there is a commitment to sustainable change throughout the organisation. However, frontline staff, feeling under resourced, are not engaged fully in the drive for continuous improvement and view it as a management activity that is being externally driven, in increasingly difficult circumstances. Policies and procedures around infection prevention are in place and while records are kept, poor communication means they are not effectively utilised. As one person put it to us:

*‘I think the organisation has everything in place … It wants to improve … But this doesn’t always filter through to our department’*.

Interviewees further spoke of a continuous turnover in casual nursing staff upsetting the localised culture and rhythms of the hospital ward:

*‘(The wards) are running on agency staff. And agency staff work in different places … So you’re always going to need to, not re-educate, but like need to tell them what to do. We’re frantically trying to tell agency staff what to do. It’s hard to make any changes or move forward in any way. We are chasing our tails’*.

Thus in addition to the potential for less familiarity of the localised practices and procedures of the hospital ward, the use of casual nursing staff may further increase the workload of permanent staff who, as well as dealing with their existing workload, need to supervise and support agency nurses.

According to Thomson, individuals cannot be held accountable for actions and omissions done in ‘ignorance’, including ‘the formal and informal expectations of the individual’s official role’ [37]. However across both hospital sites, infection prevention standards that individuals should meet, whether of practice or conduct, were not always clear to them, that official standards were distorted by the structure and culture of localised practice, and that on occasions, competing perceptions of safe practice were being played out. As Giddens observes, formal guidance plays an ambivalent and unstable role as a source of standards for practice [38]. Of particular note concerned staff awareness of the relevant rules in that certain infection prevention policies changed too frequently and that there are too many specific protocols. Focus group interviewees reported that it was simply impossible to keep up to date partly due to cuts in training, e-learning, and time and capacity for regular team meetings. This includes domestic cleaning staff who reported that their induction training had been shortened in time, by a third. Moreover, in recent times, the Health Board has implemented a new colour coding patient isolation door signing system, for different forms of infection transmission precautions. These are:

Red Sign - Contact precautions and should be used for patients in contact isolation,

Blue Sign - Droplet / airborne precautions and should be used for patients in droplet / airborne isolation, and

Yellow Sign - Protective isolation and should be used for patients requiring protective isolation.

However, a number of staff we spoke with mentioned that they were either unaware of or confused by the new door signs:

*‘Well, I didn’t know until the other day that there’s different ones. There’s some that are like blue. There’s red. I had no idea they meant different things. I just knew the patient was being isolated’*.

In part, this lack of awareness arises in that the colour coding system is being inconsistently implemented across the hospital:

*‘I’ve never seen different colours. Not on our ward, at least. You see, this is how different places (wards) are doing things differently’*.

In summary, Hospital Site A embodies a mixed patient safety culture. We found an organisation that was engaged in promoting an enhanced patient safety culture by designing strategy and structure that guide safety processes. While the structural and political domains include both positive and negative elements, the hospital embodies a cohesive cultural vision that includes a strong commitment to patients and their safety. Nonetheless, there are some underlying structural and political tensions, including poor communication, which make commitment to change, continuous improvement and infection prevention practices associated with isolation precautions difficult.

### Hospital site B

Hospital Site B is perhaps best characterised as being highly fragmented. The focus groups found significant structural issues including an absence of and mistrust in effective leadership, coupled to ever increasing time and resource pressures leading to work intensification; which are identified as being major barriers to the effective safe and appropriate care of patients and efficient infection prevention practices. The adverse nature of the structural and political context in which people at the hospital work has contributed to a cultural and emotional void; leaving staff feeling overwhelmed, depleted and demoralised. As one interviewee put it to us:

*‘(W)e’re on this constant treadmill … (And) we’re being pushed down … No matter what it is (we’re) doing, (we) struggle’*.

And another:

*‘(We’re) all working harder. That leads to more stress and yes, we probably make mistakes. That’s the culture (we’re) (working) in’*.

The structural and political context is best described as being entrenched. In recent times, staffing cuts have been made across the hospital, impacting all departments. With financial cuts, staffing shortages and increasing time pressures, the capacity to take on new initiatives, including training or education and e-learning, is limited. As one medical consultant mentioned:

*‘Training (at this hospital) is a low priority. If I applied for any training, and if I put down £300, it will never get sanctioned’*.

This leaves staff with the feeling of being unsupported by management; that they are not trained effectively and are not able to keep up to date. This was further reflected in staff evaluations and what training there is available, is that required by government. Nevertheless, this has been further compromised. A phlebotomist who has worked at the hospital for fourteen years, puts it this way:

*‘We can’t provide five or six days training to a new phlebotomist, anymore. Everything has to be done within two days ... Of course you’ve got all SOPs (Standard operating procedures), all procedures in place, but we haven’t got time and we haven’t got enough staff to give that equivalent training to people ... So infection prevention and patient safety are not as good as they once were. I don’t know how to put it in words’*.

In this way, infection prevention and patient safety in terms of governance does not always translate into practice and the challenges in making clear what is expected of people falls beyond formal standards. What staff view themselves as being responsible for is shaped by both organisational context and localised cultural norms. Focus group interviewees habitually identified gaps between what they were supposed to do and available resources for achieving this; pointing towards staffing, time pressures, access to resources and advice, management and finances. Nursing staff spoke of frequently having to double-up their responsibilities with cleaning on the wards. Hotel services talked of their frustrations in not being able to clean effectively or deep clean as infection prevention policies state, through official means. This is due to increasing time pressures, financial constraints and its impact in terms of changes in equipment and the cleaning chemicals used, and of the ambiguity of management towards effective cleaning. This has escalated in recent times, without being resolved. Moreover, stretched staff do not always know where to access resources or advice. For example, one interviewee spoke of a needlestick injury that happened the previous day and of staff not knowing how the reporting of such incidents works in practice. It was thus unclear as to whether individual wards have practices in place that address such issues. The views expressed suggest that staff perceive the process of incident reporting as being controlled by another entity within the organisation rather than a process that is owned by their hospital ward. Among those interviewees who understood the practices involved in incident reporting, completing incident report forms was perceived as a time-consuming activity that is challenging to fit into an already busy schedule. In these circumstances, staff face the dilemma of prioritising between filling in forms and caring for patients.

The focus groups confirmed the collective nature of healthcare to patient safety and infection prevention. However, on other occasions, a particular individual’s efforts are essential to preventing harm. At Hospital Site B, a staff nurse spoke of an occasion where a cleaning domestic member of staff noticed that a bed had not been cleaned to the expected standard of the hospital bed team. This followed the moving of a patient the previous evening. When the cleaning domestic member of staff queried this with the ward sister the following morning, it was identified through the paper bedding system that the bed had not been cleaned at all. In this way, the cleaning domestic staff member contributed to the prevailing conditions for patient and staff safety through observing the everyday cultural norms and standards domestic cleaning staff produce and reproduce, and through their behaviour and demonstration of their professional values.

In summary, Hospital Site B appears to have a decidedly negative context of patient safety culture: Lack of a cohesive culture and vision, coupled with a critical staffing shortage, lack of resources and emotional exhaustion contribute to this, engendering feelings of mistrust and misunderstanding between staff and senior management; each impacting on infection prevention and isolation practices. While staff struggle with focussing on effective infection prevention practice and the safe care of patients, this is not sufficient for Hospital Site B to overcome structural, political cultural and educational challenges within the organisation; for the building of a positive patient safety culture. In many ways, staff at Hospital Site B are working against ever increasing barriers.

## Discussion

This study has found two DGHs positioned against the wider economic field of ever increasing uncertainty; where struggles around effective infection prevention practices and the safety of patients are played out on the hospital wards. The utilisation of focus group interviews has revealed themes that characterises the ways in which interviewees understand the work they perform and the broader structural, political and cultural context in which they perform that work. The focus groups expose interesting features of patient safety culture and infection prevention practice within hospital isolation settings and identify important structural, political, cultural, emotional and educational challenges at each hospital site.

In a number of ways, Hospital Site A and Hospital Site B are very different places in regard to the levels of maturity of patient safety culture. Positioned against economic restructuring and wider structural and political challenges, Hospital Site A has a largely positive cultural and emotional context. That is, the hospital has a largely unified culture focused on patient care and an engaged organisation offering effective support and strong relationships; that appears especially conducive for building a mature patient safety culture. The strong emotional commitment to patients has, in a number of ways, established a shared vision for the site with staff members working together in making noticeable improvements to infection prevention practice and to the wider healthcare environment; thus ensuring patients are cared for safely and appropriately. This vision involves staff sharing knowledge that is shaped by their habitus and experiences of working in the field of healthcare [19, 28, 29, 40] and bridging cultures within teams and across departments; often in challenging circumstances.

The mantle of economic uncertainty appears to be being felt most acutely at Hospital Site B, with the uncertainties of restructuring impacting on patient safety. At this hospital, focus group interviewees continually returned to cuts in staffing and spoke in terms of the safety of patients being at a ‘very high risk’. As one staff nurse told us, ‘(W)e are so low on the ground that jobs are not being done to the standard they should be. We are falling short’. These realities appear persistent, leaving healthcare in Wales in a ‘dangerous and precarious state’ [39]. That change is being implemented primarily in response to external economic forces, appears typical for the hospital. Hospital Site B can, in many ways, be described as having a broken structural, political, educational, emotional and cultural context. At Hospital Site B, our study identified a number of issues identified in previous reports [1–5, 13, 14] and recent international studies [40–42]. These include a culture of critical staffing shortages, lack of time and resources, work intensification, a weak cultural identity, poor learning resources supporting healthcare improvement and a lack of effective organisational leadership meaning a near breakdown of relationships between staff and senior management; that appear especially challenging for the development of a mature culture of patient safety.

Habitus is a product of history; a set of values internalised by actors in processes of socialisation, where an individual’s life experiences solidify thoughts and actions into ‘durable dispositions’ that guide their future behaviours [43]. For the healthcare staff we spoke with, it offers a means of framing the world or ‘sub-field’ of patient safety [26]. From our focus group interviews, the collective nature of healthcare and the extent to which effective infection prevention practices and the culture of patient safety depends on the utilisation of cultural and social capital [25] from many different hands, became increasingly clear. For patients to remain safe, multiple interacting networks of valued relations at ward level and across departments need to go right. In spite of the increasing challenges found at both hospital sites and especially at Hospital Site B, there appears a strong cultural and emotional engagement of staff in what they view collectively as being fundamental to the work they perform. Time and again the people we spoke with returned to the shared values they viewed as being crucial to healthcare. They spoke of honesty and compassion in nursing, of the protection of dignity and respect toward patients and the safe care of patients, of competency in forming professional relationships with members of staff across grades and departments, of autonomy in making decisions, and of good infection prevention practices, including good hygiene and cleanliness. Determining a preferred course of action in healthcare requires an individual to employ legitimated knowledge or cultural capital [25], that is the accumulation of knowledge, behaviours and skills, and staff repeatedly spoke of drawing on these in their practice. In many instances and at Hospital Site B in particular, simply learning and playing by the rules while putting procedures into effect is insufficient [37, 38]. In these circumstances, it is through valued collaborative efforts of staff and informal initiatives outside of the rules that appropriate and effective safe care is delivered to patients; most often in varying and difficult conditions. These initiatives, shaped by experience and valued social relations, are drawn from taken for granted understandings and legitimated knowledge within their own occupational identity, and which they can control. That is from this group of workers whose habitus coheres with the rhythms and regularities of healthcare [19, 27–29]. Importantly, this includes the low status dirty work performed by largely invisible hospital cleaning domestic members of staff. Thus regardless of the logics of the wider economic field and conditions of uncertainty in which they work due to economic restructuring, keeping things safe for patients is critical to nursing staff and other healthcare workers. In these circumstances, hope lies in these people returning to the shared values they view as being critical to healthcare and mentioned above. As one interviewee put it to us, ‘(T)he patient comes first. We do our best. There is nothing else we can do at the moment. We simply need more staff’.

### Study strengths and limitations

To our knowledge, this is the first study to draw on the theoretical insights of Bourdieu in exploring the relationship between infection prevention practice and patient safety culture. While the research team has sought to minimise the limitations of this study, a number of important limitations need to be considered. While the study was undertaken at two hospital sites, both are located within a single health board. The lead infection prevention nurse at each hospital site identified members of staff who they subsequently contacted and invited to participate in the study. It is likely that staff members known to hold positive attitudes to infection prevention were invited to participate. However, the research design and approach to sampling enabled the authors to explore the breadth of views and knowledge held by different members of staff across occupational groups and levels of seniority.

In writing this paper, our intent was to better understand the ways in which engagement of workers with infection prevention principles and practices, shape and inform patient safety culture within the context of hospital isolation settings; and vice-versa. In a number of ways, this can only be uncovered through in-depth analysis; rather than to generalise findings from our study sample to all health boards and hospitals. Qualitative research methods are ideal for analysing the form of detailed information required to fully understand how different contexts and settings operate [36]. As such analyses are resource intensive, we chose to study two hospital sites within one health board in detail rather than do less in-depth analysis with a larger sample. Nonetheless, the richness of the information provided and discussions around infection prevention practices, levels of patient safety culture at each hospital site and the collective nature of healthcare, and of the value of Bourdieu’s theoretical insights to infection prevention and patient safety culture, provide important insights for healthcare managers in promoting, designing and implementing effective interventions where complex issues such as patient safety culture and infection prevention are involved. This is particularly important given the significant scrutiny and reports of shortcomings regarding the safety of patients and poor quality of care provided by the NHS over the last two decades [1–5, 13, 14].

### Implications for research and practice

As mentioned previously, this research, to our knowledge, is the first in exploring the relationship between the practices of infection prevention and patient safety culture that draws on the ideas of Bourdieu, and the first to theorise patient safety culture as a space of social struggle. This paper is the first paper drawn from our wider study exploring the complex issues identified here that utilises in depth interviews with patients and their relative / informal carer, healthcare workers and periods of observation on wards at both hospital sites. This study signifies the need for qualitative research methods to better understand complexities and less visible aspects of infection prevention practice and patient safety culture, especially as much previous research seeks to measure safety culture within healthcare settings [18].

This study offers an important contribution to the broader understanding of the deeper complexities of infection prevention practice and patient safety culture. The findings from this study may be used in the further design of interventions and programmes where improvements in the quality of patient care are to be made.

## Conclusion

Knowledge, awareness and understanding of infection prevention and patient safety culture within hospital isolation settings is crucial to promoting and delivering safe, appropriate and effective care to patients within the NHS. In theorising and positioning patient safety as a space of social struggle, this paper illustrates the complexities of patient safety culture. The engagement of nursing staff and other healthcare workers with infection prevention and patient safety initiatives, procedures and practices takes place in complex hospital environments and in circumstances where time and resources are most often stretched. Where work of one form is relentlessly squeezed by other demands.

This study confirms the collective nature of healthcare to patient safety and infection prevention, as well as emphasising the individual efforts of those people working within nursing and wider healthcare in preventing harm. Positioned within a context of healthcare restructuring, both hospital sites are at very different places in regard to levels of patient safety culture maturity. Hospital site A has a largely positive cultural and emotional context. The mantle of uncertainty appears to be impacting most acutely at Hospital Site B, with the uncertainties of the restructuring process impacting on the safety of patients. The largely broken structural, political, emotional and cultural context identified at Hospital Site B, appears especially challenging for the development of a mature patient safety culture.

The value of Bourdieu’s insights to infection prevention and patient safety culture lie in their focus on everyday practice. Bourdieu’s notions of field, capital and habitus reveal the taken for granted intricacies of the work nursing staff and other healthcare workers perform and position them within the wider economic field. Bourdieu offers a means of capturing the less visible aspects of healthcare. Understanding the habitus of this group of people may make it easier to design effective interventions where complex issues such as patient safety culture and infection prevention are involved.

## Data Availability

The datasets used and / or analysed during the current study are available from the corresponding author on reasonable request.

## Footnotes

1 During the submission of this paper, NHS England announced an independent review into maternity services at Nottingham University Hospitals NHS Trust. The announcement follows a report by the Care Quality Commission that rated maternity services at Nottingham University Hospitals NHS Trust as being ‘inadequate’ for a second time in two years. The review is to be Chaired by Donna Ockenden. Donna Ockenden led the independent review of maternity services at the Shrewsbury and Telford Hospital NHS Trust.

## Abbreviations

NHS: National Health Service
HIE: Hypoxic ischaemic encephalopathy
PPE: Personal protective equipment
HCAI: Healthcare-associated infection
TBP: Transmission-based precaution
DGH: District General Hospital
